# Clinical Trajectories Associated with Pharmacological Treatment Patterns in Female Adolescents with Anorexia Nervosa: A 12-Month Retrospective Study in Italian Real-World Clinical Practice

**DOI:** 10.3390/jcm15145644

**Published:** 2026-07-18

**Authors:** Pamela Fantozzi, Beatrice Fossati, Fabio Apicella, Vittorio Belmonti, Francesca Ditaranto, Raffaella Tancredi, Pietro Muratori, Valentina Levantini, Sara Calderoni

**Affiliations:** 1 IRCCS Stella Maris Foundation, Viale del Tirreno 331, 56128 Pisa, Italy; 2 Department of Clinical and Experimental Medicine, University of Pisa, 56126 Pisa, Italy

**Keywords:** anorexia nervosa, adolescence, pharmacological treatment patterns, real-world study

## Abstract

**Background**: Pharmacological treatments are frequently prescribed for adolescents with anorexia nervosa (AN), mainly to address psychiatry comorbidities and associated clinical features, although evidence supporting medication-specific effects remains limited. This retrospective study examined 12-month clinical trajectories associated with different pharmacological treatment patterns in a cohort of female adolescents with AN admitted to a tertiary university hospital. **Methods**: Clinical records of 102 female patients aged 12–18 years with AN were retrospectively reviewed. Participants were first classified according to whether they received any pharmacological treatment and were subsequently divided into six groups: no medication, antidepressants, antipsychotics, mood stabilizers, combination therapy, and medication switching. Body mass index (BMI), Clinical Global Impression—Severity (CGI-S), Children’s Global Assessment Scale (C-GAS), and item 40 of the Structured Interview for Anorexic and Bulimic Syndromes—Expert Form (SIAB-EX) were evaluated at admission (T0, before treatment initiation), at 6-month follow-up (T1), and at 12-month follow-up (T2). **Results**: BMI improved significantly over time across all treatment groups. Compared with participants who received any form of pharmacological treatment, the non-medicated group exhibited a significantly greater increase in mean CGI-S scores and significantly greater reductions in mean C-GAS and SIAB-EX scores. Baseline comparison among the six treatment groups showed significant differences in CGI-S, C-GAS, and SIAB-EX item 40 scores, indicating that the groups were not fully comparable in terms of initial clinical severity, global functioning, and physical hyperactivity. Patients in the medication-switching group generally showed smaller improvements, whereas findings in the mood-stabilizer group should be interpreted cautiously because of the very small sample size. **Conclusions**: In this real inpatients cohort, adolescents with AN showed clinical improvement over 12 months across different pharmacological treatment patterns. However, baseline differences between groups and the non-randomized retrospective design preclude causal conclusions regarding medication-specific effectiveness. These findings should be interpreted as descriptive clinical trajectories associated with treatment patterns selected in routine care. Further prospective studies with standardized treatment data and adequately powered subgroups are needed.

## 1. Introduction

Anorexia nervosa (AN) is a severe and often chronic eating disorder, with an incidence peak in adolescence [1,2]. AN is associated with a high prevalence of psychiatric comorbidity, significant resistance to treatment, and the highest mortality rate among psychiatric disorders [3,4].

According to the Diagnostic and Statistical Manual of Mental Disorders, Fifth Edition, Text Revision (DSM-5-TR) [5], AN is defined by (i) the restriction of energy intake relative to requirements, leading to a significantly low body weight; (ii) the intense fear of gaining weight or becoming fat, or persistent behaviors that interfere with weight gain; and (iii) disturbance in the way one’s body weight or shape is experienced, undue influence of body weight or shape on self-evaluation, or a persistent lack of recognition of the seriousness of their current low body weight. The DSM-5-TR distinguishes between the restrictive type (AN-R), where weight loss is achieved through caloric restriction, fasting, and/or excessive physical activity, and the binge-eating/purging type (AN-B/P), where restrictive behaviors are accompanied by binge eating or purging behaviors (e.g., self-induced vomiting, use of laxatives or diuretics) [5]. The severity of the disorder is assessed using the body mass index (BMI) for adults or the BMI-for-age percentile for children and adolescents [5,6]. Among compensatory behaviors, compulsive exercise is particularly prevalent in anorexia nervosa (AN), affecting up to 80% of individuals with the disorder. This behavior may serve to regulate or prevent negative emotional states and to facilitate weight control [7,8,9]. Psychiatric comorbidity is highly common in AN and is considered the norm rather than the exception, encompassing mood disorders, anxiety disorders, obsessive–compulsive disorders, personality disorders, and other neurodevelopmental conditions [10,11]. Furthermore, difficulties in emotion regulation are frequently observed in individuals with AN, with greater impairments being associated with increased eating disorder severity [12].

Epidemiological studies on AN have documented a recent increase in its incidence. Notably, hospital admissions related to AN rose both during [13] and after [14] the COVID-19 pandemic, with the most pronounced increases observed among pediatric populations. The lifetime prevalence of AN has been estimated at 4% in females and 0.3% in males [15]. Although the peak age of onset in both sexes has traditionally been reported between 15 and 19 years [16], a recent review highlighted a growing incidence among children younger than 15 years, which may reflect either earlier detection or a genuine shift toward an earlier age of onset [15]. Consistent with international findings, Italy’s first nationwide epidemiological survey on feeding and eating disorders reported incidence rates comparable to those observed in other countries, particularly with respect to the decreasing age of onset [17].

International guidelines identify psychological interventions, together with nutritional rehabilitation and medical stabilization, as the cornerstone of AN treatment. Although pharmacological therapies are frequently used in clinical practice, no medication has been approved for the treatment of the core symptoms of AN, particularly in children and adolescents [18,19,20,21,22]. While several studies have investigated the use of pharmacological agents in AN, findings remain inconclusive regarding their effectiveness in promoting weight restoration, and only limited evidence supports their benefit in addressing psychiatric comorbidities [18,21,23].

Regarding antidepressant medications, selective serotonin reuptake inhibitors (SSRIs), including fluoxetine, fluvoxamine, and sertraline, have been the most extensively investigated pharmacological treatments for adolescents with AN. However, evidence indicates that these agents provide little to no significant benefit in promoting weight gain or reducing associated psychopathology, such as depressive and obsessive–compulsive symptoms [24]. One proposed explanation for the limited efficacy of fluoxetine is the state of malnutrition commonly observed in AN, particularly the reduced dietary intake of tryptophan, the precursor of serotonin [23]. To address this hypothesis, a double-blind study evaluated tryptophan supplementation as an adjunct to fluoxetine treatment compared with placebo. Nevertheless, the intervention failed to demonstrate significant improvements in either weight restoration or reduction in anxiety and obsessive–compulsive symptoms [25].

Among second-generation antipsychotics (SGAs), olanzapine is one of the most extensively investigated medications for adolescents with AN. Available evidence suggests that it may contribute to improvements in anxiety, depressive, and obsessive–compulsive symptoms; weight restoration; and motor hyperactivity [26,27,28]. A recent systematic scoping review examining the use and tolerability of antipsychotics in children and adolescents with AN also identified a potential benefit of risperidone, aripiprazole, and quetiapine in promoting weight gain. However, due to the limited quality of the available evidence, the authors concluded that further research is needed before these medications can be routinely recommended in clinical practice [28].

Evidence supporting the use of mood stabilizers in AN remains limited. A recent case series involving children and adolescents with AN treated with lithium reported improvements in mood instability, adherence to nutritional rehabilitation, and psychomotor agitation [29]. Consistent with these findings, a recent review of mood stabilizers in eating disorders suggested that lithium treatment may be associated with enhanced weight gain in individuals with AN, although the evidence remains uncertain [30]. Regarding valproic acid, the largest available study in adolescents with AN found significant improvements in mood instability, treatment adherence, and aggressive behaviors [31].

Evidence supporting the use of pharmacological agents in the treatment of AN, particularly during childhood and adolescence, remains limited. Although several studies have investigated pharmacological interventions for AN, only a small number have examined longitudinal clinical trajectories according to different medication patterns in real-world settings [32]. In the present retrospective study, we reviewed clinical records from a cohort of female adolescent inpatients with AN and examined 12-month changes in BMI, global clinical severity, overall functioning, and excessive physical exercise across different pharmacological treatments patterns. Patients were categorized as receiving antidepressants, antipsychotics, mood stabilizers, combined pharmacotherapy, medication switching, or no medication. The aim of the study was to describe clinical trajectories associated with different treatment approaches, thereby providing further evidence on the pharmacological management of adolescent AN.

## 2. Materials and Methods

We retrospectively reviewed the clinical records of all patients diagnosed with anorexia nervosa restricting subtype (AN-R) or anorexia nervosa binge-eating/purging subtype (AN-B/P)—according to the criteria of the Diagnostic and Statistical Manual of Mental Disorders, Fourth Edition, Text Revision (DSM-IV-TR) [33], Fifth Edition (DSM-5) [34], or Fifth Edition, Text Revision (DSM-5-TR) [5]—who were consecutively admitted to the Eating Disorders Unit of IRCCS Fondazione Stella Maris, a tertiary-care university hospital in Pisa, Italy, between 2010 and 2024. Based on the pharmacological treatment received during hospitalization, patients were classified into six groups: (1) antidepressants; (2) antipsychotics; (3) mood stabilizers; (4) combined pharmacotherapy, defined as the concurrent use of antidepressants, antipsychotics, and/or mood stabilizers; (5) switching pharmacotherapy, including patients who changed treatment by switching from one medication class to another (e.g., from antidepressants to antipsychotics); and (6) no pharmacological treatment.

Inclusion criteria were: (a) a diagnosis of anorexia nervosa restricting subtype (AN-R) or binge-eating/purging subtype (AN-B/P), according to DSM-IV-TR [33], DSM-5 [34], or DSM-5-TR [5] criteria; (b) female sex; (c) aged between 12 and 18 years; (d) a score ≥ 85 on Raven’s Standard Progressive Matrices [35]; and (e) no pharmacological treatment at the time of recruitment. Participants with a history of pharmacological treatment were eligible only if they had completed a washout period of at least three months. Exclusion criteria included: psychotic symptoms; neurological disorders; visual or auditory neurosensory deficits; current or past substance abuse; medical conditions unrelated to the eating disorder; and significant clinical instability requiring continuous medical supervision, such as severe bradycardia, dehydration, or electrolyte imbalance.

All patients received multidisciplinary inpatient treatment delivered by the Eating Disorders Unit of IRCCS Fondazione Stella Maris. The standard care program included medical monitoring, nutritional rehabilitation, psychoeducational interventions, and psychological treatment. Psychological care involved individual sessions and family sessions, according to clinical needs. Pharmacological treatment was not assigned according to a study protocol but was prescribed by the treating clinicians on the basis of the patient’s psychiatric comorbidities, emotional and behavioral symptoms, clinical severity, and course during hospitalization. Because of the retrospective design and the long study period, the specific intensity, frequency, and content of psychological and psychoeducational interventions could not be systematically coded for all patients.

The study was conducted in accordance with the Declaration of Helsinki and received approval from the Regional Ethics Committee of Meyer Hospital (Florence, Italy; protocol code: TerapiaAN24; approved on 19 June 2024). Written informed consent was obtained from all participants and their parents or legal guardians prior to study participation.

Diagnostic assessments were conducted based on medical history; clinical observations; and a semi-structured interview using the Kiddie Schedule for Affective Disorders and Schizophrenia for School-Age Children—Present and Lifetime Version [36], which was administered to the parents of all participants by trained child neuropsychiatrists. Final diagnoses were established through consensus within a multidisciplinary clinical team. At the three assessment time points (T0 = admission to the unit; T1 = 6-month follow-up; T2 = 12-month follow-up), body mass index (BMI) was recorded and calculated as weight in kilograms (kg) divided by height in meters squared (m^2^), and the item 40 (“excessive physical exercise”) of the Structured Interview for Anorexic and Bulimic Syndromes—Expert Form (SIAB-EX) [37] was completed to assess the frequency and intensity of excessive exercise performed with the intention of burning calories. In addition, at each time point, a trained clinician administered the Clinical Global Impression—Severity scale (CGI-S) [38] to evaluate the overall severity of the disorder irrespective of its specific psychopathological features, and the Children’s Global Assessment Scale (C-GAS) [39] was used to assess the level of global functional impairment.

Statistical analyses were conducted using SPSS version 21.0. A one-way repeated-measures ANOVA was performed to examine symptom progression over time within the different groups according to the variables of interest. In addition, a two-way repeated-measures ANOVA was used to assess whether changes in these variables over time differed between groups.

We additionally compared baseline demographic and clinical characteristics across the six treatment groups. Given the small and unequal sample size of the groups, as well as the ordinal nature of some clinical measures, between-group baseline comparisons were performed using Kruskal–Wallis tests for continuous and ordinal variables. Diagnostic subtype distribution across treatment groups was compared using a global categorial test, albeit with cautious interpretation because of the small, expected cell counts. Effect sizes were reported descriptively using epsilon-squared for Kruskal–Wallis tests and Cramer’s V for categorical comparisons. These analyses were intended to describe baseline comparability between treatment groups and were not used to support causal inference regarding treatment effects.

## 3. Results

### 3.1. Demographic and Clinical Features of the Sample

A total of 102 adolescent females diagnosed with anorexia nervosa were recruited, including 91 with the restrictive subtype (AN-R) and 11 with the binge-eating/purging subtype (AN-B/P). Participants were aged between 12 and 18 years (mean age = 14.41 years, SD = 1.45). The mean duration of illness was 16.34 months (range = 3–70 months, SD = 12.70). At admission (T0), the mean body mass index (BMI) was 15.45 kg/m^2^ (range = 11–20 kg/m^2^, SD = 1.64).

**Figure 1:**
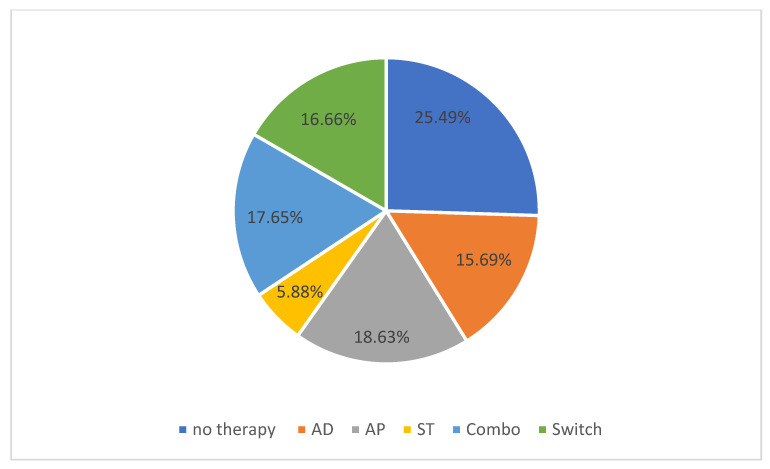
Treatments received by the six groups (AD = antidepressants; AP = antipsychotics; ST = mood stabilizers; Combo = combined medication; Switch = switching medication).

With respect to psychiatric comorbidity, depressive disorders were the most prevalent, affecting 68 patients (66.67%), followed by bipolar spectrum disorders, primarily cyclothymic disorder, in 29 patients (28.43%). Anxiety disorders were diagnosed in 38 patients (37.25%), whereas seven patients (6.86%) met the criteria for obsessive–compulsive disorders. Additionally, motor hyperactivity was documented in 63 patients (61.76%). Regarding pharmacological treatment during the follow-up period, 26 participants (25.49%) did not receive any psychopharmacological therapy, whereas 76 (74.51%) were treated with medication. Specifically, 16 subjects (15.69%) received antidepressants (AD), 19 (18.63%) antipsychotics (AP), six (5.88%) mood stabilizers (ST), 18 (17.65%) a combination of medications (Combo), and 17 (16.66%) underwent medication switching (Switch) (Figure 1). Among antidepressants, the prescribed agents were the selective serotonin reuptake inhibitors (SSRIs) sertraline, fluoxetine, and fluvoxamine. The second-generation antipsychotics used included olanzapine, aripiprazole, risperidone, and quetiapine. Regarding mood stabilizers, lithium was the most frequently prescribed agent, while valproic acid was used only in a small number of cases.

The six treatment groups did not significantly differ in age at admission, illness duration, or baseline BMI. Diagnostic subtype distribution also did not differ significantly across groups, although AN-B/P was numerically better represented in the mood-stabilizer and medication-switching groups. In contrast, significant baseline differences were observed for CGI-S, G-GAS, and SIAB-EX item 40 scores. These findings indicate that the treatment groups were not fully comparable in terms of baseline clinical severity, global functioning, and physical hyperactivity. Specifically, patients receiving combined pharmacotherapy showed higher baseline SIAB-EX item 40 scores. Descriptive characteristics of the six treatment groups are summarized in Table 1.

### 3.2. Clinical Characteristics of the Eating Disorder in Groups During the 12-Month Follow-up Based on the Treatment Received

At T0 (admission), T1 (6-month follow-up), and T2 (12-month follow-up), we compared body weight, disorder severity, global functioning, and severity of physical hyperactivity between patients who did not receive pharmacological treatment and those who underwent psychopharmacological therapy. These variables were assessed using BMI, CGI-S, C-GAS, and SIAB-EX scores (Table 2). A statistically significant increase in BMI was observed over time in both groups (*p* < 0.05). At baseline (T0), mean BMI values were comparable between the two groups. By T2, patients who did not receive pharmacological treatment exhibited a higher mean BMI than those receiving pharmacotherapy; however, this difference did not reach statistical significance. With regard to disorder severity, global functioning, and physical hyperactivity, patients who did not undergo pharmacological treatment showed significantly more favorable outcomes compared with those who received psychopharmacological therapy.

At T0, T1, and T2, the six groups were compared in terms of body weight, global functioning, and physical hyperactivity severity, which were measured using BMI, CGI-S, C-GAS, and SIAB-EX, respectively (Table 3).

**Figure 2:**
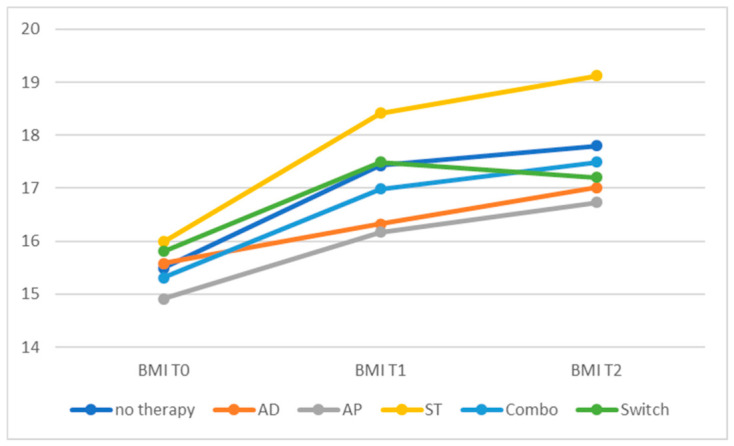
Body mass index (BMI) group comparison of patients during the 12-month follow-up (no therapy; AD = antidepressants; AP = antipsychotics; ST = mood stabilizers; Combo = combined medication; Switch = switching medication).

Over the 12-month follow-up period, a statistically significant increase in mean BMI was observed across all six treatment groups (*p* < 0.05). The mean BMI increases were 1.43 units in the antidepressant group (*p* = 0.009), 1.82 units in the antipsychotic group (*p* = 0.002), 3.14 units in the mood-stabilizer group (*p* = 0.042), 2.18 units in the combined-medication group (*p* = 0.001), 1.40 units in the medication-switching group (*p* = 0.023), and 2.31 units in the group that did not receive pharmacological treatment (*p* < 0.001) (Table 3). As illustrated in Figure 2, participants treated with mood stabilizers exhibited both the greatest increase in BMI and the highest mean BMI value at T2. In contrast, the antipsychotic group showed the lowest mean BMI values at both baseline (T0) and follow-up (T2). The smallest increases in mean BMI were observed in the antidepressant and medication-switching groups.

**Figure 3:**
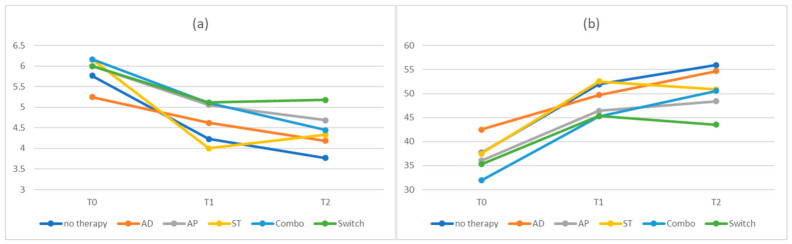
(**a**) Clinical Global Impression—Severity (CGI) and (**b**) Children Global Assessment Scale (C-GAS) group comparison of patients during the 12-month follow-up (no therapy; AD = antidepressants; AP = antipsychotics; ST = mood stabilizers; Combo = combined medication; Switch = switching medication).

All six treatment groups showed a statistically significant improvement in both overall eating disorder severity and global functioning, as assessed by CGI [38] and C-GAS [39], respectively (all *p* < 0.05). The mean reductions in CGI scores observed across the six groups were: 1.11 points (*p* = 0.001) in the antidepressant group, 1.22 points (*p* < 0.001) in the antipsychotic group, 1.84 points (*p* = 0.005) in the mood-stabilizer group, 1.73 points (*p* < 0.001) in the combined-medication group, 0.82 points (*p* < 0.001) in the medication-switching group, and 1.80 points (*p* < 0.001) in the group that did not receive pharmacological treatment (Table 3). As illustrated in Figure 3a, patients treated with mood stabilizers exhibited the greatest mean reduction in CGI scores at T2, whereas those who underwent medication switching showed the smallest decrease.

The mean increases in C-GAS scores observed across the six treatment groups were as follows: 12.65 points (*p* = 0.001) in the antidepressant group, 11.39 points (*p* < 0.001) in the antipsychotic group, 13.33 points (*p* = 0.002) in the mood-stabilizer group, 18.62 points (*p* = 0.005) in the combined-medication group, 10.24 points (*p* < 0.001) in the medication-switching group, and 18.27 points (*p* = 0.001) in the non-pharmacologically treated group. As illustrated in Figure 3b, the combined-medication group exhibited the greatest mean improvement in C-GAS scores at T2, whereas the medication-switching group showed the smallest mean increase.

**Figure 4:**
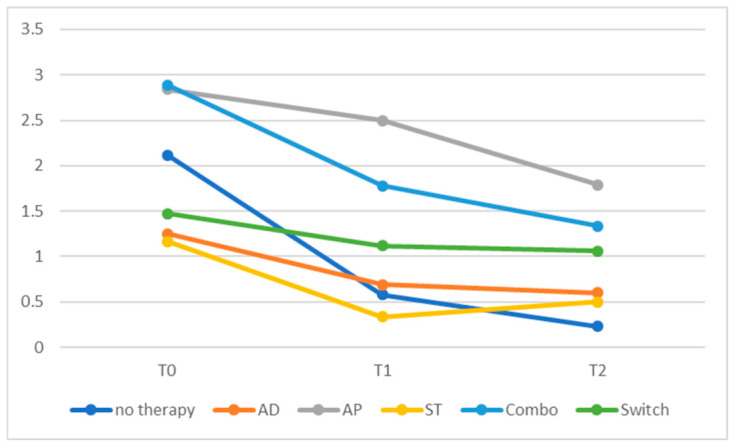
SIAB-EX group comparison of patients during the 12-month follow-up (no therapy; AD = antidepressants; AP = antipsychotics; ST = mood stabilizers; Combo = combined medication; Switch = switching medication).

Regarding physical hyperactivity, assessed using item 40 of the SIAB-EX scale [37], a statistically significant improvement was observed in three groups: participants who received no pharmacological treatment, those treated with antipsychotics, and those receiving combined pharmacotherapy (all *p* < 0.05). The mean reductions in SIAB-EX scores across the six groups were as follows: 0.69 points for the antidepressant group (*p* = 0.052), 0.94 points for the antipsychotic group (*p* = 0.012), 0.67 points for the mood-stabilizer group (*p* = 0.444), 1.56 points for the combined-medication group (*p* = 0.013), 0.41 points for the switching medication group (*p* = 0.275), and 1.89 points for the group that did not receive pharmacological treatment (*p* < 0.001) (Table 3). As illustrated in Figure 4, participants who did not receive pharmacological treatment showed the largest mean reduction in SIAB-EX scores at T2, whereas those treated with switching medication exhibited the smallest mean reduction.

## 4. Discussion

To our knowledge, this is one of the few retrospective real-world studies examining 12-month clinical trajectories (BMI, CGI-S, C-GAS, and SIAB-EX item 40) associated with different pharmacological treatment patterns in a large sample of adolescent inpatients with anorexia nervosa (AN) (n = 102). The study included patients receiving antidepressants, antipsychotics, mood stabilizers, combined pharmacotherapy, medication switching, or no medication. As the sample included all inpatients regardless of pharmacological treatment, all participants received a high level of care consisting of nutritional rehabilitation, psychoeducation, and psychotherapeutic interventions. Although current international guidelines do not recommend any specific pharmacological treatment for AN, approximately three-quarters of our adolescent inpatients were prescribed psychotropic medications to address comorbid psychiatric conditions, including depressive disorders, anxiety disorders, obsessive–compulsive disorders, and bipolar spectrum disorders.

In a recent retrospective study, Chiu et al. [32] examined BMI trajectories in a cohort of patients with AN receiving different pharmacological treatment. Compared with our sample, their cohort consisted exclusively of outpatients, included both female and male participants, and had a higher mean age. Furthermore, unlike our study, Chiu et al. did not include a group treated with mood stabilizers.

Across the overall sample, BMI, global clinical severity, global functioning, and excessive physical exercise showed improvement over the 12-month follow-up. However, because pharmacological treatment was prescribed according to clinical judgment rather than random assignment, these findings should be interpreted as longitudinal clinical trajectories observed in routine inpatient care. Although the group treated without medication achieved a higher mean BMI than the pharmacologically treated group, the difference did not reach statistical significance. However, participants who did not receive pharmacological treatment exhibited a significantly greater increase in mean CGI-S scores, as well as a significantly greater reduction in mean C-GAS and SIAB-EX scores, compared with those who received any form of pharmacological intervention. One possible explanation for this finding is that the non-medicated group may have included patients with lower clinical complexity or patients who responded sufficiently to nutritional rehabilitation, psychoeducation, and psychological treatment alone.

At the same time, evidence suggested that adherence to therapeutic recommendations may have a greater influence on the course of anorexia nervosa than the specific medication prescribed [40]. Furthermore, a strong therapeutic alliance, potentially fostered by a higher level of care, may contribute positively to patients’ clinical and functional outcomes [41].

Although the groups were comparable in terms of age, illness duration, and baseline BMI, they differed significantly in baseline CGI-S, C-GAS, and SIAB-EX item 40 scores. These differences suggest that pharmacological treatment patterns were at least partly shaped by initial clinical severity, functional impairment, and physical hyperactivity. Therefore, the observed longitudinal trajectories describe clinical courses associated with treatment patterns selected in routine clinical practice. This issue is particularly relevant for the combined-therapy and medication-switching groups, which may represent patients with more complex or severe clinical presentation.

Our study found that patients receiving antidepressant treatment experienced a significant increase in BMI 12 months after treatment initiation, consistent with previous findings [32]. In addition, the antidepressant-treated group showed a significant reduction in CGI-S scores and a significant improvement in C-GAS scores at the 12-month follow-up. These results contrast with the broader literature on the use of antidepressants in anorexia nervosa (AN), which generally reports limited evidence supporting their effectiveness in promoting weight restoration or improving psychological outcomes [24,42,43]. In the present study, antidepressant treatment consisted of three selective serotonin reuptake inhibitors (SSRIs): fluoxetine, sertraline, and fluvoxamine. Among these agents, fluoxetine is the most extensively investigated in AN and currently has the strongest evidence base supporting its use in weight-restored patients [44].

Kaye et al. [45] reported greater weight gain among patients with restricting-type anorexia nervosa (AN-R) treated with fluoxetine compared with those with binge-eating/purging-type anorexia nervosa (AN-B/P). The predominance of AN-R patients in the present sample (89.2% AN-R vs. 10.8% AN-B/P) may therefore have contributed, at least in part, to the observed findings. Consistent with this perspective, an open controlled trial examining sertraline treatment in AN-R patients found improvements in depressive symptoms, perfectionism, feelings of ineffectiveness, and impaired interoceptive awareness, although no significant effect on weight restoration was detected [46]. Overall, the mixed evidence regarding the efficacy of antidepressants in anorexia nervosa highlights the need for further research involving larger samples and longer follow-up periods.

Antipsychotic medications have been proposed as a potential therapeutic option for patients with anorexia nervosa (AN). In our study, antipsychotic treatment was associated with a significant increase in BMI after 12 months. These findings partially differ from those reported by Chiu et al. [32], who observed a significant BMI increase at 6 months but not at the 12-month follow-up. Furthermore, our sample demonstrated a significant reduction in CGI-S scores and a significant improvement in C-GAS scores after 12 months of treatment, suggestive of benefits not only in weight restoration but also in overall clinical severity and functioning. Nevertheless, the 2023 World Federation of Societies of Biological Psychiatry guidelines provide only a limited recommendation for olanzapine in AN, largely because the available evidence primarily supports its effect on weight gain [43]. While atypical antipsychotics, such as olanzapine, aripiprazole, risperidone, and quetiapine, have shown potential benefits in alleviating AN symptoms and related psychopathology among children and adolescents [24,28,47,48,49,50,51,52,53,54], the limited availability of randomized controlled trials and methodologically robust studies hampers the translation of these findings into routine clinical practice. In line with previous research demonstrating reduced activity levels in hyperactive AN patients treated with olanzapine [26], we observed a significant decrease in SIAB-EX item 40 scores, reflecting lower levels of motor hyperactivity, among participants receiving antipsychotic medication. Notably, this group had among the highest baseline SIAB-EX item 40 scores, suggesting that antipsychotics may have been preferentially prescribed to patients with greater physical hyperactivity, agitation, and clinical complexity. This pattern is clinically plausible in real-word inpatient care, but it also highlights the risk of confounding by indication.

In our sample, the group of patients treated with mood stabilizers showed a significant increase in BMI, a reduction in CGI scores, and an improvement in C-GAS scores after 12 months. However, these results should be interpreted cautiously in light of the limited sample size. The observed differences may be influenced by the small number of patients included in the analysis. For this reason, they should therefore be considered exploratory and hypothesis-generating and confirmed in larger studies. A recent review by Sesso et al. [30] reported mixed evidence regarding the efficacy of lithium and other mood stabilizers in anorexia nervosa, although lithium was associated with significant improvements in weight restoration despite some remaining uncertainty. One possible explanation is that patients treated with mood stabilizers exhibited mood fluctuations suggestive of a bipolar spectrum condition. In such cases, the stabilization of mood symptoms may contribute, first, to a reduction in overall clinical severity and, subsequently, to improvements in eating disorder psychopathology, ultimately facilitating weight gain. A growing body of research indicates that individuals with AN experience significant deficit in emotion regulation, including difficulties identifying, understanding, accepting, and managing emotional states (for recent reviews, see [55,56]). These difficulties are associated with greater eating-disorder symptom severity [57] and may represent both a risk factor for the development of AN [58] and a mechanism maintaining the disorder [59], thus representing an important treatment target [60]. 

The combined-pharmacotherapy and medication-switching groups require particular caution in interpretation, since these categories are clinically heterogeneous and may reflect complex treatment pathways. Patients receiving combined pharmacotherapy had the lowest baseline C-GAS scores, suggesting greater functional impairment at admission. At T2, this group exhibited a significant increase in BMI, a reduction in CGI-S scores, an increase in C-GAS scores, and a decrease in SIAB-EX scores after 12 months of treatment. As previously suggested, although combined therapy was generally prescribed for more severe clinical presentations, these patients appeared to benefit from the complementary effects of two medications, most commonly an antidepressant and an antipsychotic. In contrast to the findings reported by Chiu et al. [32], patients undergoing a medication switch also demonstrated a significant increase in BMI. This group additionally showed significant improvements in CGI-S and C-GAS scores. However, compared with the other treatment groups, the magnitude of improvement across outcome measures was more limited. These findings should therefore not be interpreted as evidence that switching medication led to poorer outcomes, rather switching may identify a subgroup of patients with greater illness complexity or treatment resistance. This study has several limitations that should be carefully considered. First, its retrospective observational design precludes causal inferences. Pharmacological treatments were not randomly assigned but were prescribed according to clinical judgment in routine inpatient care. Therefore, the observed clinical trajectories cannot be interpreted as medication-specific treatment effects. Second, confounding by indication represents a central limitation. Patients receiving different pharmacological treatment patterns may have differed in clinical severity, psychiatric comorbidity, emotional and behavioral instability, treatment response, and clinician-perceived need for medication; the additional baseline comparison showed significant differences among treatment groups in CGI-S, C-GAS, and SIAB-EX item 40 scores, supporting the possibility that treatment allocation was influenced by baseline clinical features. Third, some treatment subgroups were small, particularly the mood-stabilizer group. Findings from this subgroup therefore be considered exploratory and hypothesis-generating. Fourth, the available retrospective database did not include systematic information on medication dosage, treatment duration, timing of medication initiation, reason for switching, adherence, discontinuation, adverse events, or serum levels of mood stabilizers. Fifth, all patients received multidisciplinary inpatient care, including medication monitoring, nutritional rehabilitation, psychoeducation, and psychological treatment. However, because data were collected retrospectively over a long time period, it was not possible to systematically code the intensity, frequency, content, or adherence to different treatments. Sixth, although BMI is clinically relevant and widely used, raw BMI is less developmentally informative than BMI-for-age percentile, BMI z-score, or percentage of median BMI in adolescents sample. These standardized indicators could not be reliably calculated from the available retrospective database and should be included in future studies. Seventh, SIAB_EX item 40 was used to assess excessive physical exercise. Although clinically meaningful, this single item does not provide a comprehensive assessment of eating-disorder psychopathology or compulsive exercise. Eighth, the study did not include an a priori sample size calculation. The simple size was determined by the number of eligible patients admitted during the study period. Finally, the study included only female adolescents admitted to a tertiary-care inpatient unit. Therefore, the findings may not generalize to male patients, younger children, adults, outpatients, or less severe clinical populations.

## 5. Conclusions

In this retrospective real-world inpatient cohort of female adolescents with anorexia nervosa, clinical improvement over 12 months was observed across different pharmacological treatment patterns. BMI increased significantly in all groups, and improvements were also observed in global clinical severity, global functioning, and excessive physical exercise, although trajectories varied across treatment categories.

However, the study design does not allow conclusions regarding medication-specific effectiveness; pharmacological treatment was prescribed according to clinical judgment, and baseline differences between groups indicate that treatment allocation was likely influenced by clinical severity, functional impairment, and physical hyperactivity. The more favorable outcomes observed in some groups, as well as the smaller improvements observed in the medication-switching group, should therefore be interpreted as descriptive clinical trajectories rather than causal effects of treatment.

These findings contribute real-world longitudinal data on the pharmacological management of adolescent AN in inpatient care. Future prospective studies with larger samples, standardized treatment protocols, detailed pharmacological exposure data, adolescent-specific weight outcomes, and appropriate controls for baseline clinical severity are needed to clarify the role of psychopharmacological treatment in this population.

## Figures and Tables

**Table 1:** Baseline demographic and clinical characteristics of the six treatment groups.

	No Therapy n = 26	AD n = 16	AP n = 19	ST n = 6	Combo n = 18	Switch n = 17	*p* Value
Age	14.19 ± 1.63	14.44 ± 1.41	14.26 ± 1.33	15.17 ± 1.33	14.17 ± 1.42	14.41 ± 0.94	0.724
Illness duration	14.50 ± 12.37	14.56 ± 9.11	16.47 ± 13.10	17.50 ± 10.29	15.06 ± 11.59	13.76 ± 5.88	0.860
BMI (kg/m^2^)	15.49 ± 2.07	15.58 ± 1.29	14.91 ± 1.53	15.99 ± 1.96	15.31 ± 1.46	15.81 ± 1.41	0.734
CGI-S	5.77 ± 0.65	5.25 ± 0.58	6.00 ± 0.67	6.17 ± 0.75	6.17 ± 0.62	6.00 ± 0.61	0.002
C-GAS	37.69 ± 4.95	42.50 ± 5.77	36.05 ± 7.92	37.50 ± 6.89	31.94 ± 7.88	35.29 ± 8.56	<0.001
SIAB-EX item 40	2.12 ± 1.77	1.25 ± 1.69	2.84 ± 1.50	1.17 ± 1.83	2.89 ± 1.49	1.47 ± 1.84	0.015
AN-R, n (%)	25 (96.2)	16 (100.0)	16 (84.2)	4 (66.7)	17 (94.4)	13 (76.5)	0.063
AN-B/P, n (%)	1 (3.8)	0 (0.0)	3 (15.8)	2 (33.3)	1 (5.6)	4 (23.5)	0.063

AD = antidepressants; AP = antipsychotics; ST = mood stabilizers; Combo = combined medication; Switch = switching medication; BMI = body mass index; CGI-S = Clinical Global Impression—Severity; C-GAS = Children’s Global Assessment Scale; SIAB-EX = Structured Interview for Anorexic and Bulimic Syndromes—Expert Form.

**Table 2:** Mean and standard deviations of body mass index (BMI), Clinical Global Impression—Severity (CGI-S) score, Children Global Assessment Scale (C-GAS) score, and score of item 40 of the Structured Interview for Anorexic and Bulimic Syndromes—Expert Form (SIAB-EX) at T0 (admission), T1 (6-month follow-up), and T2 (12-month follow-up) in patients who did not receive pharmacological therapy and in patients treated with any pharmacological therapy.

	T0	T1	T2	F	*p* Value
**BMI** (kg/m^2^)				0.458	0.458
No pharmacological therapy	15.49 (±2.08)	17.73 (±1.86)	17.80 (±2.14)		
Any pharmacological therapy	15.43 (±1.48)	16.87 (±1.65)	17.26 (±2.12)		
**CGI-S**				5.967	0.004
No pharmacological therapy	5.77 (±0.65)	4.23 (±0.71)	3.77 (±0.91)		
Any pharmacological therapy	5.89 (±0.70)	4.89 (±0.84)	4.62 (±1.12)		
**C-GAS**				3.54	0.032
No pharmacological therapy	37.69 (±4.95)	51.92 (±6.49)	55.96 (±7.62)		
Any pharmacological therapy	36.38 (±8.23)	47.17 (±8.14)	49.21 (±9.31)		
**SIAB-EX**				6.608	0.003
No pharmacological therapy	2.12 (±1.77)	0.58 (±1.24)	0.23 (±0.82)		
Any pharmacological therapy	2.07 (±1.79)	1.45 (±1.69)	1.17 (±1.58)		

**Table 3:** Mean and standard deviation of body mass index (BMI), Clinical Global Impression—Severity (CGI-S) score, Children Global Assessment Scale (C-GAS) score, and score of the item 40 of the Structured Interview for Anorexic and Bulimic Syndromes—Expert Form (SIAB-EX) at T0 (admission), T1 (6 months follow-up), and T2 (12-month follow-up) in the six groups (no therapy; antidepressants = AD; antipsychotics = AP; mood stabilizers = ST; combined medication = Combo; switching medication = Switch).

	T0	T1	T2	F	*p* Value
**BMI** (kg/m^2^)					
No therapy	15.49 (±2.07)	17.73 (±1.86)	17.80 (±2.14)	12.016	<0.001
AD	15.58 (±1.29)	16.33 (±1.23)	17.01 (±1.44)	6.636	0.009
AP	14.91 (±1.53)	16.17 (±1.40)	16.73 (±1.38)	9.752	0.002
ST	15.99 (±1.96)	18.42 (±2.00)	19.13 (±2.78)	7.737	0.042
Combo	15.31 (±1.46)	16.99 (±1.26)	17.49 (±2.12)	12.148	0.001
Switch	15.81 (±1.42)	17.5 (±1.97)	17.21 (±2.84)	4.926	0.023
**CGI-S**					
No therapy	5.57 (±0.65)	4.23 (±0.71)	3.77 (±0.91)	62.581	<0.001
AD	5.29 (±0.59)	4.59 (±0.80)	4.18 (±0.95)	12.529	0.001
AP	6.00 (±0.69)	5.06 (±0.73)	4.78 (±1.10)	14.538	<0.001
ST	6.17 (±0.75)	4.00 (±1.10)	4.33 (±1.03)	25.000	0.005
Combo	6.17 (±0.62)	5.11 (±0.76)	4.44 (±1.20)	49.961	<0.001
Switch	6.00 (±0.61)	5.12 (±0.78)	5.18 (±1.29)	17.937	<0.001
**C-GAS**					
No therapy	37.69 (±4.95)	51.92 (±6.49)	55.96 (±7.62)	68.538	<0.001
AD	42.06 (±5.88)	50.00 (±5.86)	54.71 (±7.39)	23.103	<0.001
AP	36.11 (±8.15)	46.39 (±7.63)	47.50 (±7.52)	9.209	0.002
ST	37.50 (±6.89)	52.50 (±6.12)	50.83 (±6.65)	27.118	0.005
Combo	31.94 (±7.89)	45.28 (±9.92)	50.56 (±10.13)	26.586	<0.001
Switch	35.29 (±8.56)	45.29 (±8.38)	43.53 (±9.81)	10.406	0.001
**SIAB-EX**					
No therapy	2.12 (±1.77)	0.58 (±1.24)	0.23 (±0.86)	15.578	<0.001
AD	1.25 (±1.69)	0.69 (±1.49)	0.56 (±1.21)	3.676	*p* = 0.052
AP	2.83 (±1.54)	2.50 (±1.58)	1.89 (±1.68)	5.975	0.012
ST	1.17 (±1.84)	0.33 (±0.82)	0.50 (±1.23)	1.000	0.444
Combo	2.89 (±1.49)	1.78 (±1.73)	1.33 (±1.65)	5.787	0.013
Switch	1.47 (±1.84)	1.12 (±1.58)	1.06 (±1.64)	1.409	0.275

## Data Availability

Data are unavailable due to privacy restrictions.
